# Core Buildup Composite Resins Bond Strength to Dentin and Microhardness Using Universal Adhesives

**DOI:** 10.3390/jfb17080411

**Published:** 2026-08-18

**Authors:** Iana Schmitt, Jorge Perdigão, Guilherme Carpena Lopes

**Affiliations:** 1 Department of Dentistry, Federal University of Santa Catarina (UFSC), Florianópolis 88040, SC, Brazil; iana.schmitt@posgrad.ufsc.br (I.S.); guilherme.lopes@ufsc.br (G.C.L.); 2 Department of Restorative Sciences, Division of Operative Dentistry, University of Minnesota, Minneapolis, MN 55455, USA

**Keywords:** dentin bonding, universal adhesive, shear bond strength, microhardness, composite resin

## Abstract

The peer-reviewed literature on the use of universal adhesives (UAs) with core build-up composite resins (CBCRs) is limited. UAs may enhance the polymerization of dual-cured composite resins, leading to increased microhardness due to the “touch-cure” effect. This study compared the dentin bond strengths and microhardness of CBCRs bonded with UAs. A total of 480 bovine incisors were randomly allocated into 20 groups (*n* = 24). Two dual-cured CBCRs, ParaCore (PrCore) and Gradia Core (GrCore), one light-cured CBCR, Clearfil Photo Core (PhotoCore), and Filtek Z250 (Z250) were evaluated. For the dual-cured CBCRs, the UAs from the respective manufacturers were mixed with a dual-cure activator (Act): One Coat 7 Universal (OC7+Act) and G-Premio Bond (GrPB+Act). Three additional UAs were evaluated: Tokuyama Universal Bond II (TUB), Scotchbond Universal Plus Adhesive (SBUP), and Scotchbond Universal Adhesive with Dual Cure Activator (SBU+Act). Shear bond strength was evaluated after 24 h. Additional specimens were prepared for Vickers microhardness (VHN) evaluation, with or without UA application to the bottom surface. Bond strengths ranged from 7.4 ± 4.0 to 37.0 ± 7.5 MPa. GrCore and PrCore showed higher bond strengths with SBU+Act than with their manufacturer-recommended adhesives. Dual-cured CBCRs exhibited lower dentin bond strengths and lower VHN than the light-cured composite resins, and the touch-cure effect was more effective for GrCore and PhotoCore.

## 1. Introduction

Extensive coronal destruction presents a challenge in restorative dentistry. Given that restorations in these cases often rely solely on dentin bonding for support, core buildup procedures have become a routine solution [1,2,3]. These core buildups typically utilize composite resins with dual-curing polymerization [4,5], which offer a conservative alternative to cast or prefabricated post-and-core systems while reducing chair time and number of material interfaces [6,7]. Dual-cured core buildup composite resins (CBCRs) and other dual-cured composite resins can be light-activated in addition to their intrinsic chemical polymerization [8,9,10]. Chemical polymerization ideally ensures curing in areas with minimal or no exposure to the curing light [8,9,10,11,12]. Furthermore, single-increment or bulk insertion and curing of dual-cured composite resins offers practical advantages [13] over the incremental application required for traditional light-cured composite resins [1]. However, light-cured CBCRs remain clinically relevant due to their lower cost, wider shade availability, and established compatibility with light-cured adhesives.

UAs have become increasingly popular, primarily due to their simplified application and broad indications. Nevertheless, laboratory studies have demonstrated incompatibility between simplified adhesives and self- (or chemical-)cured and dual-cured composite resins [14,15,16,17,18,19,20,21,22,23,24]. This incompatibility can be of chemical nature, stemming from the low pH of adhesives [14,21,22], or physical, attributable to the high water permeability of the adhesive layer formed by simplified adhesive systems, which may absorb water from the underlying dentin and weaken the interface [16,17,20]. One potential cause of chemical incompatibility is the adverse interaction between incompletely polymerized acidic resin monomers in the oxygen-inhibited superficial layer and the high-pH tertiary amine catalyst in self- and dual-cured composite resins [14], including CBCRs [23,24]. Given that current UAs also exhibit acidic pH values ranging from 1.5 to 3.2, this chemical incompatibility could also arise [25,26].

Dual-cure activator solutions mixed with UAs may prevent the chemical incompatibility between simplified adhesives and self- and dual-cured composite resins [17,18,27,28,29,30,31,32]. Activator solutions often contain the sodium salt of aryl sulfinic acid to overcome this incompatibility [33,34]. Manufacturers claim that mixing UAs with their respective dual-cure activators ensures the polymerization of their dual-cured composite resins, as this reaction is chemically initiated by co-initiators (such as sodium aryl sulfinate salts) within the adhesive [35]. As a result, dual-cured CBCRs can achieve adequate bond strengths to dentin [23,36].

UAs mixed with their respective dual-cure activator initiate the polymerization at the interface when the activator in the adhesive contacts the dual-cured composite resin [35,37,38]. A recent study investigating dual-cured resin cements reported that the incorporation of touch-cure activators enhanced the degree of polymerization (or conversion) of all self-cured resin cements [39]. However, these activators did not improve the degree of conversion of the corresponding dual-cured counterparts [39].

Manufacturers have developed other strategies to address this potential incompatibility and enable the touch-cure effect. Some light-cured adhesives now incorporate a built-in dual-cure activator, in addition to traditional light-cure activators, for improved polymerization [37]. Rather than relying on a separate dual-cure activator, Scotchbond Universal Adhesive Plus (SBUP, Solventum, St. Paul, MN, USA) incorporates copper acetate monohydrate, a transition metal salt, as a dual-polymerization accelerator [37,40]. This ingredient, according to the manufacturer, improves compatibility with both dual-cured and chemically cured composite resins by catalyzing the decomposition of the peroxide component in chemically curing initiator systems. SBUP has been investigated primarily through in vitro [41,42,43] and clinical studies of light-cured composite resins [42,43,44,45,46]. Dentin bond strengths with SBUP, when used with light-cured composite resins, are comparable to those of its predecessor Scotchbond Universal Adhesive (SBU, Solventum, St. Paul, MN, USA) [43,45]. However, SBU must be mixed with its proprietary dual-cure activator for application with dual-cured composite resins, including CBCRs and some resin cements [47].

Tokuyama Universal Bond II (TUB. Tokuyama Dental, Tokyo, Japan) contains a proprietary monomer known as 3D-SR (three-dimensional self-reinforcing monomer). It forms a hydrolysis-resistant Ca-salt on dentin hydroxyapatite, which may result in extended bond durability [48]. TUB also utilizes borate-initiated chemical polymerization. The manufacturer asserts that this polymerization mechanism increases dentin bond strengths that are comparable or superior to those obtained with light-cured UAs combined with dual-cured composite resins. A recent study confirmed that TBU resulted in high dentin bond strengths when the dual-cured composite resin was light-cured [49]. Another project reported that the dentin bond strengths of TBU associated with dual-cured CBCRs were not affected by the curing mode of the CBCR (light-cured or self-cured) [50].

Bond strengths depend on adequate polymerization of both the UA and the composite resin used for core buildup [36]. The microhardness of composite resins is a reliable parameter for evaluating the efficiency of their polymerization, given its direct association with the degree of conversion [51,52]. The top-to-bottom microhardness ratio has been proposed as a simple and effective method for evaluating polymerization at different depths within composite resins [53]. Therefore, comparing microhardness at both the top and bottom surfaces of CBCRs provides insight into the potential touch-cure effect of UAs when these adhesives are applied to the surface farther from the light-curing source.

Independent studies comparing the dentin bond strengths of dual-cured and light-cured CBCRs combined with UAs with different polymerization modes are scarce. Furthermore, the degree of polymerization of these composite resins has not been adequately assessed through microhardness testing. Therefore, the primary objective of this study was to compare the dentin bond strengths of dual-cured and light-cured CBCRs, using UAs with distinct polymerization modes (light-cured, self-cured, and dual-cured). Additionally, microhardness was assessed both with and without contact of composite resins with UAs to investigate the potential touch-cure effect of UAs. A light-cured composite resin indicated for direct restorations served as control. The following null hypotheses were tested: (1) the dentin bond strengths of CBCRs are not influenced by their polymerization mode; (2) the dentin bond strengths of CBCRs are not influenced by the polymerization mode of the UA employed; and (3) the microhardness of CBCRs is not influenced by their polymerization mode and by the UAs and their potential touch-cure effect.

## 2. Materials and Methods

### 2.1. Dentin Shear Bond Strengths

A total of 480 bovine incisors were cleaned and then inspected for enamel cracks or carious lesions using a magnifying loupe (ExamVision ApS, Samsø, Denmark). The teeth were initially sectioned at the CEJ under water cooling, and the root portion was discarded. The crowns were embedded in phenolic rings with chemically curing acrylic resin, and superficial dentin was exposed by sequential grinding with silicon carbide papers up to 600-grit under water irrigation. The specimens were stored in purified water at room temperature. Labial dentin surfaces were wet-polished with 600-grit SiC paper for 60 s immediately prior to the adhesive application procedures to create a standardized smear layer [54]. Subsequently, the specimens were randomly allocated into 20 groups (*n* = 24). A perforated double-sided adhesive tape (3M) with a 2.5 mm diameter opening was used to standardize the bonding area.

All UAs were applied according to the respective manufacturers’ instructions (Table 1). The UAs included three dual-cured adhesives (OC7+Act, GrPB+Act, and SBU+Act), one self-cured adhesive (TUB), and one light-cured adhesive (SBUP). For dual-cured adhesives, the adhesive and respective activator were mixed immediately before application. Adhesives were applied to dentin in self-etch mode, air-dried, and light-cured if recommended by the respective manufacturer (Table 1).

Two dual-cured CBCRs, ParaCore (PrCore, Coltene, Altstätten, Switzerland) and Gradia Core (GrCore, GC Corp., Tokyo, Japan), and one light-cured CBCR, Clearfil Photo Core (PhotoCore, Kuraray Noritake Dental, Tokyo, Japan), were evaluated. Filtek Z250 (Z250, Solventum, St. Paul, MN, USA), a light-cured composite resin for direct restorations, served as the control. Details regarding the materials utilized, including their lot numbers, composition, classification, and application protocol, are enumerated in Table 1. Table 2 presents the experimental groups. Composite resin cylinders (2.38 mm wide × 2.0 mm high) were fabricated using a bonding assembly device (Ultradent Products Inc., South Jordan, UT, USA) and light-cured with an LED curing unit (Valo Corded, Ultradent Products Inc.) at an irradiance of approximately 810 mW/cm^2^, according to the manufacturers’ instructions. After photoactivation, the specimens were stored in purified water for 24 h at 37 °C. Shear bond strength tests were performed in a universal testing machine (Instron 4444, Instron Corp., Norwood, MA, USA) at a crosshead speed of 1.0 mm/min. Using a Linear Test Slide (Ultradent Products Inc.), a metallic notch with a diameter of 2.38 mm was positioned perpendicular to the composite resin cylinder and loaded until failure occurred at the dentin–composite interface. The mean dentin bond strengths (MPa) were calculated by dividing the load at failure (in Newtons) by the bonded surface area (in mm^2^).

### 2.2. Failure Mode

The failure modes were analyzed using an optical microscope (Leica DM4000 M, Leica Microsystems GmbH, Wetzlar, Germany) at ×40 magnification. Failures were classified as adhesive (AD), cohesive in composite resin (CR), cohesive in dentin (CD), or mixed (MI). Mixed failures were defined as the simultaneous occurrence of adhesive and cohesive failure modes within the specimen.

### 2.3. Vickers Microhardness (VHN)

Specimens were prepared using cylindrical Teflon molds (2 mm deep × 20 mm external diameter and having a 4 mm wide cylindrical opening) positioned on a glass plate (bottom side). The dual-cured CBCRs (PrCore and GrCore), the light-cured CBCR (PhotoCore), and the control composite resin (Z250) were inserted into molds in a single increment (n = 6). A Mylar matrix was positioned over the composite resin (top side). To allow composite resin excess to be extruded over the Teflon mold, the Mylar matrix was pressed using a glass plate. Furthermore, the Mylar matrix was supported by the surrounding upper surface of the mold to ensure a flat composite resin surface prior to light-activation. All materials were light-activated from the top side according to the respective manufacturer’s instructions (Table 1), removed from the molds, and stored in water in a light-proof container for 24 h.

Extra specimens were prepared to test the effect of the touch-cure effect. Each UA was applied onto a glass plate (bottom side) prior to the insertion of the composite resins (n = 6). The UAs were not light-cured before composite resin placement, and specimens were stored in water for 24 h after light activation of the composite resin from the top side.

VHN was measured using a microhardness tester (Wilson VH1102, Buehler, Lake Bluff, IL, USA) under a load of 0.98 N for 15 s. Three indentations were performed on the top and bottom surfaces of each specimen, and mean VHN was calculated from the indentation measurements obtained using the microhardness tester’s optical system.

### 2.4. Statistical Analysis

Data normality was assessed using the Kolmogorov–Smirnov and Shapiro–Wilk tests. Dentin bond strength data were analyzed with ANOVA considering the effect of “composite resin polymerization mode” and “UA polymerization mode” as independent variables. Failure types (adhesive, cohesive in composite resin, cohesive in dentin, and mixed) were quantified for pattern analysis [55]. Descriptive statistics were used to calculate the percentage of each type of failure.

VHN data were analyzed with multivariate ANOVA considering “composite resin polymerization mode”, “UA polymerization mode” and “surface side” as independent variables. A separate two-way ANOVA was carried out for the touch-cure effect by comparing VHN of each composite resin with “application of UA” and “without the application of UA” onto the bottom surface. Statistical analyses were performed using IBM SPSS 30.0 (IBM, New York, NY, USA).

## 3. Results

Normal distribution was confirmed by the Kolmogorov–Smirnov test. ANOVA demonstrated that the composite resin polymerization mode (*p* < 0.001) and the UA polymerization mode (*p* < 0.001) significantly influenced dentin bond strengths. In addition, a statistically significant interaction between these factors was observed.

The predominant failure mode was adhesive, except for the PhotoCore/TUB, Z250/TUB, and Z250/SBUP groups, for which mixed failures were predominant.

Overall, PhotoCore and Z250 resulted in statistically higher mean bond strengths than the dual-cured CBCRs PrCore and GrCore. For the combinations of UAs and composite resins, the lowest statistical ranking of mean bond strengths included the two dual-cured CBCRs: PrCore/OC7+Act (7.4 MPa); PrCore/GrPB+Act (7.9 MPa); PrCore/SBUP (8.5 MPa); GrCore/GrPB+Act (8.8 MPa); GrCore/OC7+Act (11.6 MPa); and PrCore/TUB (11.7 MPa) (Table 3). The combination of OC7+Act with PrCore, as recommended by the respective manufacturer, resulted in statistically lower bond strengths than those obtained when PrCore was used with SBU+Act. For GrCore, the mean bond strengths with the respective manufacturer-recommended GrPB+Act were statistically lower than those obtained with the adhesives TUB, SBU+Act, and SBUP. Z250/SBUP and PhotoCore/OC7+Act resulted in statistically higher mean bond strengths than all the other groups (*p* < 0.05, Table 3). The adhesives GrPB+Act, TUB, and SBUP resulted in statistically higher bond strengths when combined with the two light-cured composite resins (PhotoCore and Z250) than with the two dual-cured CBCRs (PrCore and GrCore) (*p* < 0.05) (Table 3).

Regarding the VHN, the factor “composite resin polymerization mode” exerted a significant influence on mean VHN (*p* < 0.001), as the light-cured composites resulted in statistically higher mean VHN than the dual-cured CBCRs both at the top surface and the bottom surface without application of UA. For each composite resin, the mean VHN of the top surface was statistically higher than that of the bottom surface without UA (*p* < 0.05) (Table 4). PrCore exhibited the highest top-to-bottom without UA ratio (1.46), while the other three composite resins resulted in similar top-to-bottom without UA ratios that ranged from 1.21 to 1.26 (Table 4). The four composite resins resulted in statistical differences among them regardless of the surface side (top versus bottom surfaces without UA) (Table 4). Z250 resulted in the highest mean VHN of all composite resins at both the top and bottom surfaces without UA applied to the bottom surface, whereas PrCore resulted in the lowest mean VHN of all composite resins at both the top and bottom surfaces, both with and without UA applied to the bottom surface (*p* < 0.05).

When UA was applied to the bottom surface to test for the touch-cure effect, OC7+Act combined with the respective manufacturer-recommended PrCore resulted in statistically similar mean VHN compared with the VHN obtained without UA (Table 4). On the other hand, the combination of GrPB+Act with the respective dual-cured GrCore resulted in a significant increase in mean VHN at the bottom surface compared with GrCore without UA. The contact with TUB did not statistically change the mean VHN of the two dual-cured CBCRs at the bottom surface but increased the mean VHN of PhotoCore. For the light-cured composite, Z250, there was no difference between the mean VHN at the bottom surface when it was not in contact with an UA compared to the mean VHN in contact with an UA, except for when OC7+Act was used. In general, the mean VHN of GrCore and PhotoCore increased with the application of UA on the bottom surface (Table 4).

Similar to the mean VHN obtained without the application of UA to the bottom surface, the highest top-to-bottom VHN ratio when UA was applied to the bottom surface was obtained when PrCore was used in conjunction with the manufacturer-recommended OC7+Act (Table 4).

## 4. Discussion

The present study revealed that the light-cured CBCR (PhotoCore) and the light-cured composite resin used for direct restorations (Z250) resulted in higher mean dentin bond strengths compared to dual-cured CBCRs. The compatibility of different UA polymerization modes with the two light-cured composite resins was unexpected. Specifically, the self-cured (TUB), dual-cured (GrPB+Act), and light-cured (SBUP) modes each yielded superior mean bond strengths when used with PhotoCore and Z250, in contrast to their performance with the dual-cured CBCRs. This apparent inconsistency, particularly regarding TUB and GrPB+Act, may be ascribed to the accelerated polymerization rate and concomitant temperature elevation observed within the two light-cured composite resins. This phenomenon could expedite the chemical curing mechanism of the two adhesives. Consequently, the performance of the tested composite resins was influenced by their respective curing modes, as well as by the curing modes of some of the UAs. Therefore, the first null hypothesis was partially rejected.

Chemical incompatibility between simplified adhesives and dual-cured CBCRs can interfere with polymerization at the adhesive interface. Our study specifically found that the performance of dual-cured CBCR was UA-dependent. This suggests that the interaction between UAs and dual-cured CBCRs varies based on the formulation of the products, corroborating other reports [23,32,55]. Although previous studies have reported bond strengths above 20 MPa for OC7+Act/PrCore [23,55], our findings revealed poorer performance for this manufacturer-recommended combination. The two dual-cured CBCRs, PrCore and GrCore, combined with SBU+Act, resulted in higher mean bond strengths compared with those obtained with the two proprietary dual-cured UAs recommended for the same CBCRs (OC7+Act and GrPB+Act, respectively). Although OC7+Act and GrPB+Act contain similar well-established redox accelerator systems for the dual-cure activation [56,57], their performance was inferior to that of SBU+Act. The lower dentin bond strengths observed for dual-cured CBCRs in conjunction with their manufacturer-specified dual-cured UAs bear clinical significance. The potential for incompatibility between simplified, low-pH adhesives and self- or dual-activated composite resins may compromise the degree of polymerization [4,17,21], thereby potentially reducing the longevity of the resultant core build-up restoration.

Similar incompatibility has been reported for GrPB+Act associated with dual-cured CBCRs, and inclusion of dual-cure activators did not effectively solve this problem [58]. Collectively, these findings reinforce the need to evaluate the compatibility between CBCRs and different UAs rather than relying exclusively on manufacturer-recommended combinations. The chemical characteristics of the components present in the composition of some UAs and/or the respective activator may render the UAs incompatible with the respective dual-cured CBCRs, regardless of the curing mode. OC7+Act resulted in higher mean bond strengths in conjunction with the light-cured CBCR PhotoCore (34.8 ± 6.5 MPa). This combination also resulted in relatively high VHN compared with the other UAs tested, further supporting the significant influence of the CBCR on the bonding effectiveness of this UA.

Among the UAs tested with the light-cured composite resin Z250, SBUP exhibited the highest mean dentin bond strengths. These findings are consistent with those of Tsujimoto et al. [59], who reported a mean shear dentin bond strength of 34.6 (±6.5) MPa using the same SBUP with a light-cured composite resin. Furthermore, SBUP has demonstrated excellent clinical performance when utilized with light-cured composite resins [60]. However, the present study observed lower dentin bond strengths for SBUP compared to its predecessor SBU+Act when combined with the two dual-cured CBCRs (Table 3). The presence of copper acetate monohydrate in SBUP may offer a more limited strategy to overcome chemical incompatibility with dual-cured CBCRs compared to SBU+Act, which may be more related to the latter’s touch-cure effect. Nevertheless, Sinhoreti et al. [61] reported an intrinsic touch-cure mechanism for SBUP, evidenced by its capacity to enhance the polymerization of a dual-cured resin cement under dark conditions. This phenomenon likely arises from an interaction between the metal salt initiator within SBUP and the peroxide component of the resin cement. This potential touch-cure effect of SBUP may account for the observed increase in mean VHN on the bottom surface of GrCore (65.3) relative to the same surface without the UA (49.9) (Table 4). Conversely, this potential touch-cure effect with GrCore did not manifest at the base of the other dual-cured CBCR, PrCore, despite the shared presence of tertiary amines in both CBCRs. No touch-cure effect was observed for OC7+Act when combined with the manufacturer-recommended PrCore. This conclusion is based on the finding that this specific combination did not yield statistically significant differences in mean VHN, irrespective of whether OC7+Act was applied to the bottom surface. Furthermore, PrCore exhibited the highest average top-to-bottom VHN ratio in the absence of UA application to the bottom surface. This suggests a considerably higher degree of conversion at the light-cured top surface compared to the bottom surface, which relied solely on the material’s self-curing properties. Analysis of the mean top-to-bottom ratio with UA applied to the bottom surface (Table 4) further revealed that PrCore consistently resulted in the highest average top-to-bottom VHN ratio. This indicates a potential concern regarding the degree of conversion on the surface farther from the light-curing source, particularly when in contact with UA. The respective manufacturer recommends light-curing a 2 mm layer of PrCore for 20 s. As our VHN specimens were 2 mm high, further research into increased light-curing time for this material is warranted. Also, the results suggest that inserting and light-curing PrCore in increments thinner than 2 mm would likely yield better mechanical properties—a finding that requires confirmation in future studies. Additionally, future research should evaluate this top-to-bottom ratio for PrCore using LED light-curing units with irradiance levels higher than the currently recommended 800 mW/cm^2^. In fact, there are other reasons for clinicians to insert PrCore with an incremental technique. For example, Chan et al. [62] reported that the shrinkage strain of PrCore was significantly higher than that of a light-cured composite resin. Moreover, they observed a decrease in volumetric shrinkage when the material was applied in two 2 mm increments, as opposed to a single 4 mm increment.

Conversely, the mean top-to-bottom ratio for GrCore with UA applied to the bottom surface was 1.0 (Table 4), implying a uniform degree of conversion at varying depths for this dual-cure CBCR in spite of the shorter light curing time of 10 s recommended by the respective manufacturer. While these findings related to dual-cure CBCRs may not be directly extrapolatable to a clinical scenario, clinicians may need to consider them.

The discrepancy between the behavior of PrCore and GrCore suggests that variations in monomer composition, initiator concentration, co-initiator/inhibitor systems, and other formulation-related factors may have affected the efficacy of the touch-cure mechanism [63]. These factors include the presence of Bis-GMA as the main methacrylate molecule in PrCore, while UDMA serves the same role in GrCore. The viscosity of Bis-GMA is 100 times higher than that of UDMA [64], requiring the use of more TEGDMA in PrCore as a diluent for Bis-GMA. TEGDMA also has very high water sorption [64], which may have interfered with the activation mechanism of the light-cured SBUP. In addition, higher concentrations of UDMA in composite resins result in higher polymerization rate and degree of conversion of light-cured resin-based materials as compared to resins rich in Bis-GMA [65]. This may also explain the higher VHN for GrCore at the top and bottom surfaces without UA when compared to PrCore. Beyond these intrinsic differences between the CBCRs, the differences observed among material combinations may be related to the characteristics and compatibility of the redox initiation systems involved in the polymerization process. Despite the slightly higher mean VHN at the base surface of GrCore in contact with SBUP compared to that of GrCore in contact with SBU+Act, the two UAs had an opposite effect on the dentin bond strengths of GrCore. This finding suggests that bottom surface microhardness alone does not fully explain the dentin bond strength results. Factors related to polymerization at the adhesive–composite resin interface, such as the compatibility between the polymerization initiation systems of the UA and the CBCR, may also have contributed to the differences in dentin bond strengths [14]. Therefore, future studies should investigate whether the addition of a separate dual-cure activator improves the bonding performance of SBUP when used with dual-cured CBCRs [61].

The VHN was influenced by the composite resin polymerization mode, leading to the rejection of the second hypothesis. Light-cured composite resins exhibited a higher mean VHN compared to dual-cured CBCRs. Furthermore, a comparison of the mean VHN without UA applied to the bottom surface to the corresponding VHN with UA contact revealed that VHN was influenced by the composite resin polymerization mode, the UAs, and their potential touch-cure effect. This evidence leads to a partial rejection of the third hypothesis.

TUB, a self-cured UA, may elicit a touch-cure effect due to the borate salts in its composition generating free radicals, which enhance the chemical polymerization of the adhesive even in the absence of light [50]. Borate salts improve the degree of conversion of adhesives by forming salt-acid binaries that initiate free radical polymerization [49]. The high bond strengths observed for TUB in conjunction with GrCore may be attributed to the interfacial compatibility of this UA with dual-cured CBCRs rather than to a touch-cure effect, as TUB exhibited the lowest VHN when in contact with GrCore. Overall, the touch-cure effect of the UAs tested was somewhat limited and exhibited inconsistency among the composite resins utilized in this study. The touch-cure effect may assume greater importance in clinical scenarios for some materials involving deeper or thicker increments, where the curing light energy may not adequately reach the innermost regions of the tooth. Therefore, future research is warranted to evaluate this effect in thicker composite resin increments.

PhotoCore and Z250 exhibited more homogeneous VHN behavior, which suggests efficient polymerization in depth compared to the dual-cure CBCRs. Although a previous study did not observe significant differences in dentin bond strengths between light-cured and dual-cured CBCRs [63], the present study demonstrated improved performance for the light-cured composite resins. This finding may be associated with a higher degree of conversion, which has been reported to improve both microhardness and bond strength [51,52]. Consistent with this explanation, PhotoCore and Z250 groups showed the highest means for both properties, dentin bond strength and VHN. The results also demonstrated that the type of CBCR exerted greater influence on VHN, corroborating the literature indicating that composition and inorganic filler content are determinants of mechanical properties [63].

The association between VHN and dentin bond strengths suggests that polymerization efficiency plays a fundamental role in the performance of composite resins, including CBCRs, although significant interactions among the factors indicate that microhardness behavior varied according to composite resin and specimen surface side. Further research should include other commercially available materials to provide more comprehensive information to clinicians.

The present study had some limitations: (1) It tested 2 mm high specimens to measure VHN. However, clinicians typically place buildup restorations on root-filled teeth that are deeper than 2 mm. The lower VHN observed for some materials in this study may be even more critical in deeper restorations. For instance, PrCore might benefit from smaller increments and incremental light polymerization rather than solely relying on its dual-cure potential. (2) Another limitation of this study is the VHN test specifically designed to investigate the potential touch-cure effect by maximizing the interaction between the UAs and CBCRs under standardized laboratory conditions. Therefore, although the findings provide mechanistic insight into adhesive–composite resin compatibility, further microhardness studies using dentin substrates are necessary to confirm its clinical relevance; and. (3) Our study did not include thermocycling. According to one report, thermocycling decreased the dentin bond strengths when SBUP was applied in self-etch mode in conjunction with a dual-cured resin cement [66]. The same study also reported a reduction in bond strengths for TUB after thermocycling.

## 5. Conclusions

Based on the findings of this study, we can draw the following conclusions:

1. Dual-cured CBCRs resulted in lower dentin bond strengths compared to light-cured CBCR and a composite resin used for direct restorations.
2. Under the conditions evaluated in this study, not all dual-cured universal adhesives demonstrated optimal compatibility with their corresponding dual-cured CBCR. Therefore, clinicians should exercise caution when using specific product combinations.
3. The VHN top-to-bottom ratio demonstrated that not all dual-cured CBCRs achieve an acceptable degree of polymerization in areas not in contact with the light-curing source.
4. Both light-cured composite resins, PhotoCore and Z250, exhibited higher VHN than the dual-cured CBCRs.
5. The “touch-cure” effect of the tested universal adhesives in contact with dual-cured CBCRs appears to be limited.

## Figures and Tables

**Table 1:** Materials used in this study, lot numbers, compositions, classifications, and application protocols.

Materials (Lot Numbers)	Manufacturers	Compositions	Classifications	Application
ParaCore (PrCore) N21447 Dentin shade	Coltene, Altstätten, Switzerland	Bis-GMA, TEGDMA, UDMA, TMPTMA, NaF, Ba-glass, amorphous silica, initiators, accelerators	Dual-cured core buildup composite resin	Light-curing time (>800 mW/cm^2^): 20 s per side/surface for a 2.0 mm thick layer. The total time between adhesive application and cementation should not exceed 5 min
Gradia Core (GrCore) 2404220 Universal shade	GC Corp., Tokyo, Japan	UDMA, NPGDMA, GDMA, silanated Al–F-silicate glass (70–75%), amorphous silica, TiO_2_, Fe_2_O_3_, MgO, initiators, accelerators	Dual-cured core buildup composite resin	>700 mW/cm^2^ for 10 s. Can be applied in layers up to 2.5 mm thick. Allow the material to self-cure for 5 min if light cannot reach the material
Clearfil Photo Core (PhotoCore) B70307	Kuraray Noritake Dental, Tokyo, Japan	Silanized silica filler, Silanized barium glass filler, Bisphenol A diglycidyl methacrylate (Bis-GMA), Triethylene glycol dimethacrylate (TEGDMA), Benzoyl peroxide, dl-Camphorquinone	Light-cured core buildup composite resin	Light intensity above 300 mW/cm^2^. It should be light-cured (with LED) for 20 s in layers up to 4.0 mm
Filtek Z250 (Z250) 602A2 Shade A2	Solventum, St. Paul, MN, USA	Bis-GMA, UDMA, Bis-EMA, TEGDMA, non-agglomerated zirconia/silica particles	Light-cured composite resin	It should be light-cured with an LED unit at a minimum intensity of 400 mW/cm^2^. For increments up to 2.5 mm thick, light-cure for 20 s
One Coat 7.0 Universal (OC7) N07006 pH = 2.8	Coltene, Altstätten, Switzerland	Methacrylates, photoinitiators, ethanol, water	Light-cured universal adhesive	
One Coat 7.0 Activator (OC7+Act) N23390	Coltene, Altstätten, Switzerland	Ethanol, N,N-bis(2-hydroxyethyl)-p-toluidine, water, and activators	Dual cure activator for One Coat 7.0 Universal	Dispense a fresh drop of One Coat 7 Universal and one drop of One Coat 7.0 Activator and mix thoroughly with a clean disposable brush (approximately 5–10 s). Apply the adhesive mixture to dentin using a disposable brush for 20 s. Gently air-dry for 5 s using oil-free compressed air. Light cure for 10 s with a light intensity greater than 800 mW/cm^2^
G-Premio Bond (GrPB) 2404220 pH = 1.51	GC Corp., Tokyo, Japan	10-methacryloyloxydecyl dihydrogen phosphate (10-MDP), phosphoric acid ester monomer, acetone, phosphorylated ethyl methacrylate, 4-methacryloyloxyethyl trimellitate, dimethacrylate, initiator, and silicon dioxide	Light-cured universal adhesive	
G-Premio Bond DCA (GrPB +Act) 2404221	GC Corp., Tokyo, Japan	Initiator, distilled water, 2,2′-(4-methylphenylimino) bisethanol, and ethanol	Dual cure activator for G-Premio BOND	Mix G-Premio BOND and G-Premio BOND DCA in a 1:1 ratio and apply to the prepared post space. Allow it to react for 20 s. Apply the adhesive with a disposable brush. Wait for 10 s. Air-dry for 5 s using maximum air pressure. Light cure for 10 s
Tokuyama Universal Bond II (TUB) 032E44 pH = 2.2–2.8	Tokuyama Dental, Tokyo, Japan	Liquid A: Phosphoric acid monomer (3D-SR monomer), MTU-6, HEMA, Bis-GMA, TEGDMA, acetone, and others. Liquid B: γ-MPTES, borate, peroxide, acetone, ethanol, water, and others	Self-cured universal adhesive	Dispense one drop each of Tokuyama Universal Bond II A and B into the disposable mixing well. Mix thoroughly with a disposable applicator until the mixed bonding agent turns green. Complete the application within 1 min after dispensing when using the mixing well, as Tokuyama Universal Bond II contains volatile solvents. Air-dry within 30 s after application to ensure the proper film thickness. Dry until the still-fluid Tokuyama Universal Bond II remains in place without any movement
Scotchbond Universal Adhesive Plus (SBUP) 11108491 pH = 2.7	Solventum, St. Paul, MN, USA	10-methacryloyloxydecyl phosphate, 1,3-benzenediol 2-(2-hydroxyethoxy) ethyl 3-hydroxypropyl diethers, 2-hydroxyethyl methacrylate, 2-methyl-2-propenoic acid, 3-(triethoxysilyl)propyl ester, reaction products with silica, APTES, ethanol, water, camphorquinone, acrylic and itaconic acid copolymer, and copper acetate monohydrate	Light-cured universal adhesive	Apply with agitation for 20 s. Air-dry for at least 5 s until the adhesive no longer moves. Light-cure for 10 s.
Scotchbond Universal Adhesive (SBU) 2503000256 (also referred to as Single Bond Universal in certain countries) pH = 2.7	Solventum, St. Paul, MN, USA	10-MDP phosphate monomer, methacrylate-modified polyalkenoic acid copolymer, HEMA, dimethacrylate resins, silane, initiators, ethanol, and water	Light-cured universal adhesive	Self-etch mode: Rub the adhesive for 20 s; air-blow for 5 s to thin the layer; light cure for 10 s.
Scotchbond Universal Dual Cure Activator (SBU+Act) 11651743	Solventum, St. Paul, MN, USA	Phosphoric acid ester monomers, HEMA (2-hydroxyethyl methacrylate), ethanol, water, camphorquinone, N,N-dimethylbenzocaine, sodium p-toluenesulfinate, and silica filler	Dual cure activator (DCA) for Scotchbond Universal Adhesive	Place one drop of Scotchbond Universal Adhesive and one drop of Scotchbond Universal DCA into a mixing well and mix for 5 s. Use a disposable applicator to apply the mixture to the entire tooth structure and rub for 20 s. Then gently air-blow the liquid for approximately 5 s until it no longer moves and the solvent has completely evaporated. Light cure the adhesive with a conventional curing light for 10 s.

Bis-GMA = Bisphenol A diglycidylmethacrylate; TEGDMA = triethyleneglycol dimethacrylate; UDMA = urethane dimethacrylate; TMPTMA = Trimethylolpropane trimethacrylate; NPGDMA = Neopentyl glycol dimethacrylate; GDMA = Glycol Dimethacrylate; Bis-EMA = bisphenol A-diglycidyl methacrylate ethoxylated; MTU-6 = 6-methacryloxyhexyl 2-thiouracil-5-carboxylate, HEMA = 2-hydroxyethyl methacrylate; γ-MPTES = 3-(triethoxysilyl) propyl methacrylate; APTES = 3-(aminopropyl)triethoxysilane.

**Table 2:** Groups formed from the combinations of composite resins and universal adhesives used in the study.

Groups	Composite Resins	Universal Adhesive
PrCore/OC7+Act	ParaCore	One Coat 7.0 Universal mixed with One Coat 7.0 Activator
PrCore/TUB	ParaCore	Tokuyama Universal Bond II
PrCore/SBUP	ParaCore	Scotchbond Universal Adhesive Plus
PrCore/GrPB+Act	ParaCore	G-Premio Bond mixed with G-Premio Bond Dual-Cure Activator
PrCore/SBU+Act	ParaCore	Scotchbond Universal Adhesive mixed with Scotchbond Universal Dual Cure Activator
GrCore/OC7+Act	Gradia Core	One Coat 7.0 Universal mixed with One Coat 7.0 Activator
GrCore/TUB	Gradia Core	Tokuyama Universal Bond II
GrCore/SBUP	Gradia Core	Scotchbond Universal Adhesive Plus
GrCore/GrPB+Act	Gradia Core	G-Premio Bond mixed with G-Premio Bond Dual Cure Activator
GrCore/SBU+Act	Gradia Core	Scotchbond Universal Adhesive mixed with Scotchbond Universal Dual Cure Activator
PhotoCore/OC7+Act	Clearfil Photo Core	One Coat 7.0 Universal mixed with One Coat 7.0 Activator
PhotoCore/TUB	Clearfil Photo Core	Tokuyama Universal Bond II
PhotoCore/SBUP	Clearfil Photo Core	Scotchbond Universal Adhesive Plus
PhotoCore/GrPB+Act	Clearfil Photo Core	G-Premio Bond mixed with G-Premio Bond Dual-Cure Activator
PhotoCore/SBU+Act	Clearfil Photo Core	Scotchbond Universal Adhesive mixed with Scotchbond Universal Dual Cure Activator
Z250/OC7+Act	Filtek Z250	One Coat 7.0 Universal mixed with One Coat 7.0 Activator
Z250/TUB	Filtek Z250	Tokuyama Universal Bond II
Z250/SBUP	Filtek Z250	Scotchbond Universal Adhesive Plus
Z250/GrPB+Act	Filtek Z250	G-Premio Bond mixed with G-Premio Bond Dual-Cure Activator
Z250/SBU+Act	Filtek Z250	Scotchbond Universal Adhesive mixed with Scotchbond Universal Dual Cure Activator

**Table 3:** Mean dentin shear bond strengths * (MPa ± SD).

Composite Resin	Universal Adhesive	Mean Bond Strengths * (MPa ± SD)
ParaCore	OC7+Act	7.4 ± 4.0 ^a^
	TUB	11.7 ± 5.1 ^a^
	SBUP	8.5 ± 3.3 ^a^
	GrPB+Act	7.9 ± 3.6 ^a^
	SBU+Act	18.9 ± 4.8 ^bc^
Gradia Core	OC7+Act	11.6 ± 6.7 ^a^
	TUB	17.2 ± 8.4 ^b^
	SBUP	16.5 ± 11.8 ^b^
	GrPB+Act	8.8 ± 6.2 ^a^
	SBU+Act	26.8 ± 8.8 ^d^
Clearfil Photo Core	OC7+Act	34.8 ± 6.5 ^e^
	TUB	27.2 ± 9.4 ^d^
	SBUP	22.6 ± 3.4 ^bcd^
	GrPB+Act	25.4 ± 7.0 ^cd^
	SBU+Act	23.4 ± 6.9 ^cd^
Filtek Z250	OC7+Act	24.8 ± 9.5 ^cd^
	TUB	26.9 ± 6.8 ^d^
	SBUP	37.0 ± 7.5 ^e^
	GrPB+Act	21.6 ± 5.3 ^bc^
	SBU+Act	24.0 ± 9.1 ^cd^

SD = standard deviation. * Means with the same superscript letter are not statistically different (*p* < 0.05).

**Table 4:** Mean VHN number * ± SD.

Composite Resin	Top Surface and Bottom Surface Without UA	Bottom Surface with UA Applied onto the Bottom Surface		Top/Bottom Ratio with UA Applied onto the Bottom Surface	Mean Top/Bottom Ratio with UA Applied onto the Bottom Surface
ParaCore	Top surface = 53.8 ^C^ (±3.3) Bottom surface = 36.9 ^A^ (±4.5) (top/bottom ratio = 1.46)	OC7+Act	35.0 ^a^ (±3.2)	1.54	1.44
		TUB	40.2 ^c^ (±4.4)	1.33	
		SBUP	35.4 ^a^ (±3.2)	1.51	
		GrPB+Act	39.6 ^bc^ (±3.2)	1.35	
		SBU+Act	36.4 ^ab^ (±4.0)	1.47	
Gradia Core	Top surface = 60.3 ^D^ (±4.0) Bottom surface = 49.9 ^B^ (±3.6) (top/bottom ratio = 1.21)	OC7+Act	58.7 ^e^ (±2.5)	1.02	1.02
		TUB	52.1 ^d^ (±4.3)	1.16	
		SBUP	65.3 ^f^ (±4.3)	0.92	
		GrPB+Act	59.9 ^e^ (±5.5)	1.00	
		SBU+Act	60.5 ^e^ (±4.1)	0.99	
Clearfil Photo Core	Top surface = 80.7 ^G^ (±4.8) Bottom surface = 63.7 ^E^ (±5.3) (top/bottom ratio = 1.26)	OC7+Act	70.6 ^gh^ (±7.3)	1.14	1.14
		TUB	71.5 ^gh^ (±3.0)	1.12	
		SBUP	69.0 ^gh^ (±4.2)	1.17	
		GrPB+Act	72.2 ^h^ (±6.1)	1.11	
		SBU+Act	69.1 ^fgh^ (±4.4)	1.16	
Filtek Z250	Top surface = 88.4 ^H^ (±4.3) Bottom surface = 72.6 ^F^ (±6.3) (top/bottom ratio = 1.22)	OC7+Act	65.2 ^f^ (±2.3)	1.35	1.29
		TUB	69.0 ^fgh^ (±2.3)	1.28	
		SBUP	67.6 ^fg^ (±2.0)	1.30	
		GrPB+Act	71.1 ^gh^ (±4.2)	1.24	
		SBU+Act	69.1 ^fgh^ (±3.3)	1.27	

SD = standard deviation. * Means with the same superscript letter are not statistically different (*p* < 0.05). Lowercase letters are associated with mean VHN with application of UA to the bottom surface.

## Data Availability

The original contributions presented in this study are included in the article. Further inquiries can be directed to the corresponding author.
